# Structure-function relationships of wheat flavone *O*-methyltransferase: Homology modeling and site-directed mutagenesis

**DOI:** 10.1186/1471-2229-10-156

**Published:** 2010-07-29

**Authors:** Jian-Min Zhou, Eunjung Lee, Francesca Kanapathy-Sinnaiaha, Younghee Park, Jack A Kornblatt, Yoongho Lim, Ragai K Ibrahim

**Affiliations:** 1 Plant Biochemistry Laboratory and Centre for Structural-Functional Genomics, Concordia University, Montreal, QC, H4B 1R6, Canada; 2 Division of Bioscience and Biotechnology, BMIC, RCD, Konkuk University, Seoul 143-701, Korea; 3 Enzyme Research Group, Department of Chemistry & Biochemistry, Concordia University, Montreal, QC, H4B 1R6, Canada

## Abstract

**Background:**

Wheat (*Triticum aestivum *L.) *O*-methyltransferase (TaOMT2) catalyzes the sequential methylation of the flavone, tricetin, to its 3'-methyl- (selgin), 3',5'-dimethyl- (tricin) and 3',4',5'-trimethyl ether derivatives. Tricin, a potential multifunctional nutraceutical, is the major enzyme reaction product. These successive methylations raised the question as to whether they take place in one, or different active sites. We constructed a 3-D model of this protein using the crystal structure of the highly homologous *Medicago sativa *caffeic acid/5-hydroxyferulic acid *O*-methyltransferase (MsCOMT) as a template with the aim of proposing a mechanism for multiple methyl transfer reactions in wheat.

**Results:**

This model revealed unique structural features of TaOMT2 which permit the stepwise methylation of tricetin. Substrate binding is mediated by an extensive network of H-bonds and van der Waals interactions. Mutational analysis of structurally guided active site residues identified those involved in binding and catalysis. The partly buried tricetin active site, as well as proximity and orientation effects ensured sequential methylation of the substrate within the same pocket. Stepwise methylation of tricetin involves deprotonation of its hydroxyl groups by a His262-Asp263 pair followed by nucleophilic attack of SAM-methyl groups. We also demonstrate that Val309, which is conserved in a number of graminaceous flavone OMTs, defines the preference of TaOMT2 for tricetin as the substrate.

**Conclusions:**

We propose a mechanism for the sequential methylation of tricetin, and discuss the potential application of TaOMT2 to increase the production of tricin as a nutraceutical. The single amino acid residue in TaOMT2, Val309, determines its preference for tricetin as the substrate, and may define the evolutionary differences between the two closely related proteins, COMT and flavone OMT.

## Background

The structural diversity of flavonoid compounds in plants is the result of a number of enzyme-catalyzed substitution reactions [1]. Of these, enzymatic *O*-methylation is mediated by a family of substrate-specific, position oriented *O*-methyltransferases (OMTs; EC 2.1.1-). Substrate methylation confers significant changes to the physiochemical properties of methyl acceptor molecules by altering their solubility, reactivity and interaction with cellular targets. Several plant OMTs have been characterized both at the biochemical and molecular levels [2], and most of the enzymes involved in flavonoid biosynthesis, including flavonoid OMTs, were recently studied at the structural level [3].

**Figure 1:**
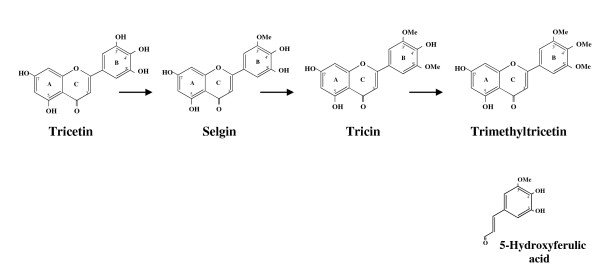
**Pathway for the sequential methylation of tricetin**. The structure of 5-hydroxyferulic acid shows the analogy with the flavonoid B-ring and its 3-carbon tail of selgin.

Wheat (*Triticum aestivum *L.) flavone OMT (TaOMT2) catalyzes the sequential methylation of the flavone, tricetin (5,7,4'-trihydroxy-3',5'-dimethoxyflavone) to its 3'-monomethyl- (selgin), 3',5'-dimethyl- (tricin) and 3',4',5'-trimethyl ether derivatives (Fig. 1), although tricin appears to be the major (*ca*. 95%) enzyme reaction product [4]. Examples of multiple methylations have been reported mostly for the *N*-methyltransferases (NMTs), phosphoethanolamine-specific NMT in mammalian cells [5,6] and wheat leaves [7], as well as the conserved SET domain of Rubisco large subunit NMT [8] and viral histone lysine NMT [9]. In contrast, the enzymatic synthesis of pentamethylated flavonols in *Chrysosplenium americanum *(Saxifragaceae) leaves has been shown to be catalyzed, in a stepwise manner, by a number of OMTs which exhibit distinct regiospecificities and physiochemical properties [10], as well as characteristic kinetic properties [11].

TaOMT2 and its analogues constitute a distinct flavone OMT gene family that has recently been characterized in a number of cereal species, including rice [12], barley and maize [13], as well as a few other graminaceous species (Additional file 1). In fact, alignment of their amino acid sequences shows that the putative residues involved in substrate binding and catalysis are strictly conserved [13]. Several members of this OMT family have previously been mis-annotated as caffeic acid/5-hydroxyferulic acid 3/5-OMTs (COMTs), possibly because of their high amino acid sequence similarity/identity to flavonoid OMTs and the structural analogy between their phenylpropanoid moiety and the flavonoid B-ring with its 3-C side chain (Fig. 1). In fact, TaOMT2 accepts 5-hydroxyferulic acid (5HFA) as an alternate substrate with ~78% relative activity, but with > 2-fold lower affinity and a 4-fold lower turnover than its preferred substrate, tricetin [4].

There are > 100 functionally diverse MTs that have been structurally characterized [3,14], including several plant OMTs [15-17] that belong to Class I structures. Regardless of the level of overall sequence identity, these enzymes share a common conserved *S*-adenosyl-L-methionine (SAM) binding domain with a core α/ß Rossman fold [18] and a unique α-helical cap that forms the top of the active site cavity. Except for the mammalian catechol OMT [19] and caffeoyl CoA OMT [20] whose reaction mechanisms invoke a divalent cation-dependent process, that of other OMTs is thought to proceed *via *direct transfer of the SAM-methyl group to the substrate with inversion of symmetry in a S_N_2-like mechanism [21] and the removal of a proton before, during or after methyl transfer [22]. The fact that TaOMT2 catalyzes the methylation of three different substrates: tricetin, selgin and tricin (Fig. 1) raised the question as to whether this enzyme protein possesses one substrate binding pocket for the three substrates, or three different sites.

To circumvent the difficulties we encountered in obtaining high quality crystals, we resorted to homology modeling of TaOMT2 using *Medicago sativa *MsCOMT [16] as a template; the two proteins share 63% sequence identity. In addition, the fact that the reaction product of COMT, 5HFA, is structurally similar to ring B and its 3-C side chain of selgin, the first methylated intermediate of TaOMT2 (Fig. 1), suggests a close evolutionary relationship between these two enzyme proteins.

The aim of this article was to study the architecture of the active site of TaOMT2 in relation to the sequential methylation of tricetin, and propose a mechanism whereby the single protein can catalyze three successive methylations. Furthermore, the proposed structural model allowed us to investigate the role of Val309 in defining the substrate preference of TaOMT2.

## Methods

### Chemicals

Most phenolic compounds used in this study were from our laboratory collection. Tricetin was purchased from Indofine Chemical Company (Hillsborough, NJ). Its methylated derivatives were synthesized by the condensation of 2,4,6-trihydroxyacetophenone with a suitably substituted benzaldehyde to give rise to the corresponding flavanone, followed by dehydrogenation with iodine and NaOAc [23]. Identity of the methylated products was verified by NMR and mass spectroscopy. [^3^H]SAM (80 Ci/mmol) was purchased from American Radiolabeled Chemicals (St. Louis, MO). Unless otherwise specified, all other reagents were of analytical grade.

### Homology modeling and molecular docking

The 356 amino acid sequence of TaOMT2 was obtained from GenBank (Accession number ABB03907). For homology modeling, the 2.2 Å resolution X-ray structure [16] of MsCOMT (1KYZ.pdb) was used as the template. The sequences of the two proteins are 63% identical, their substrates are structurally analogous, and the reactions they catalyze are methylations of similar compounds mediated by SAM as the co-substrate. Based on the above considerations, there is a good reason to believe that MsCOMT is a good template for TaOMT2. There are three chains (A, E and C) that are readily seen in the unit cell of MsCOMT even though the protein in solution, like TaOMT2, is probably a dimer [16]. The A and E chains form a tight dimer interface, whereas the dimeric complement to the C chain is not visible. It is essential to point out that even though the dimer of TaOMT2 is undoubtedly the functional unit in solution, monomers are also catalytically active. All three chains contain the reaction products S-adenosyl-L-homocysteine (SAH) and 3-(4-hydroxy-3-methoxyphenyl)-2-propenoic acid.

The modeled three dimensional structure of TaOMT2 was built using SWISS-MODEL http://swissmodel.expasy.org. The template consisted of the 360 residues visible in the crystal structure of the MsCOMT E-chain; the polyhistidine tag and the first five residues are not visible. The monomeric structure of TaOMT2, 356 residues omitting the first seven residues, was constructed using the MsCOMT-E chain as a template. All molecular modeling experiments were done with SYBYL (Tripos, St. Louis, MO; http://www.tripos.com), except for calculations of the solvated protein (see below). The secondary structure of the modeled TaOMT2 was determined using the method of Kabsch and Sander provided in Sybyl. Sybyl calculations were carried out on an Intel Core 2 Quad Q6600 (2.4 GHz) Linux PC workstation. The initial structure obtained from SWISS-MODEL was subjected to energy minimization (EM) and molecular dynamics (MD) [24]. Gasteiger-Huckel charge was used to determine the 3-D structures of the ligands: tricetin, selgin, and tricin, which were subjected to EM by the conjugate gradient algorithm using the Tripos Force Field; these are the most probable structures in solution but are probably distorted in the actual protein. For solvation, the protein was embedded in a 5Å shell of 10,942 water molecules, and the protein-water complex was transferred into Sybyl.

Since MsCOMT contains SAH as a co-factor, its location in TaOMT2 was determined by superimposing the TaOMT2 on MsCOMT. Tricetin, selgin, and tricin were docked manually into TaOMT2 using FlexX Single Receptor Module in Sybyl. Residues Met123, Asn124, Phe169, Met173, Val309, Ile312, Met313 and Asn317 were assigned as the binding site for docking as reported for MsCOMT [16]. The selection radius for automatic docking was 6.5 Å, and the docking process was iterated 30 times per ligand. The final structure was chosen on the basis of the docking score and presumed to represent the spatially correct docking. The docking score for tricetin ranged between -19.03 and -12.48, and the docking model with a score of -15.71 was fitted to the binding site and used in this study.

The homodimeric structure of TaOMT2 was generated using the Biopolymer Module-Align Structure in the Sybyl program. 1KYZ.PDB contains no hydrogens; they were introduced into our modeled TaOMT2 using the H-bonds Module in Sybyl. The cutoff for H-bonds was a minimum of 3 Å between appropriate atoms. The size of the binding pocket was measured using the Docking Simulation Module in Sybyl. The secondary structures of TaOMT2 as well as the mutant proteins were determined using Biopolymer Display Module in Sybyl. The structural models generated in this study were viewed in PyMOL http://pymol.sourceforge.net or Sybyl.

### Site-directed mutagenesis

TaOMT2 cDNA mutants were prepared using QuickChange site-directed mutagenesis kit (Stratagene, CA) and sequenced before subcloning into the expression vector. Since the MsCOMT cDNA clone was not available, the putative *Medicago truncatula *MtCOMT EST cDNA clone (NF035B09NR, GenBank Accession No. AW686202; Noble Foundation, Inc., Ardmore, OK), was used instead. MsCOMT and MtCOMT share 98% identity at the amino acid level. The wild type cDNAs in vector pET200/D-TOPO were used as templates for PCR.

Primers used for generating the mutants are shown in Additional file 2.

### In vitro protein expression, enzyme assays and kinetic analyses

After sequencing, the mutant plasmids were transformed into *E. coli *BL21 (DE3) cells (EMD, Darmstadt, Germany) for protein expression. The recombinant proteins were purified to near homogeneity by affinity chromatography on a Ni-NTA column (Qiagen, Mississauga, ON). SDS-PAGE was used to check purity of the recombinant proteins, and the highly purified fractions were stored at 4°C until used.

The standard enzyme assays were performed as previously described [4] using 50 μM of the phenolic substrate, 50 μM SAM containing 25 nCi of radioactive label, and 0.1 to 2.0 μg of the affinity-purified recombinant protein.

Kinetic analyses were performed using 1.8 μg of the affinity-purified proteins with a saturating concentration of SAM, containing 25 nCi of radioactivity, and varied concentrations (5 μM to 50 μM) of the phenolic substrates. Assays were performed in triplicates and were repeated twice. Lineweaver-Burk plots were applied for the determination of *K*_*m*_, V_max _and K_cat _values [25].

## Results

### Structural model of TaOMT2

**Figure 2:**
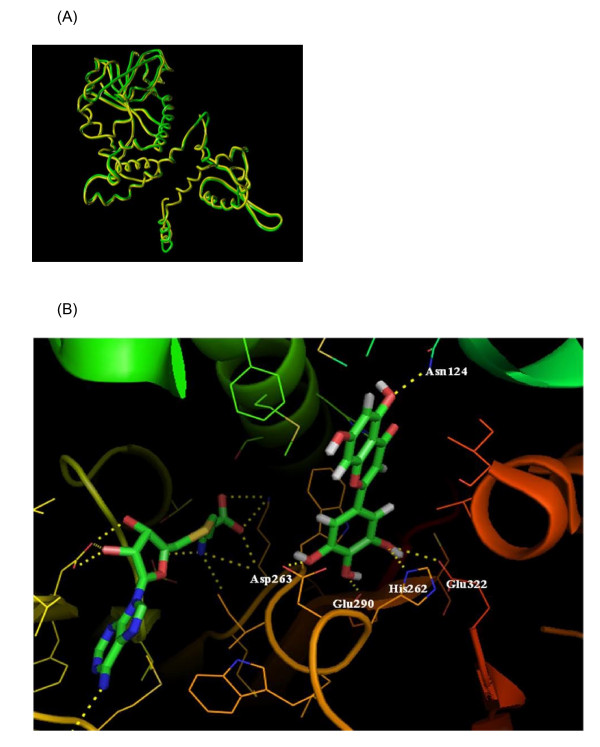
**Proposed structure of TaOMT2**. **A**, Superposition of the backbones of both proteins - shown in ribbon form with an RMSD value of 0.9 Å. MsCOMT is colored green and TaOMT2 yellow; B, Active site of TaOMT2. A 3-D structure showing the H-bond network among SAH, tricetin and their neighboring residues. H-bonds are within a distance of 1.53Å to 2.11Å and are shown in dotted lines.

The overall structure of TaOMT2 is that of a compact dimer with a 21% overlap of the two monomers, and an N-terminal helix that contributes to the dimer interface. The fact that the amino acid sequence of TaOMT2 exhibits a 63% identity to the previously crystallized MsCOMT (PDB, 1KYZ) [16] and have structurally similar substrates, allowed us to construct a reliable 3-D structure of the target protein using the latter as a template. This rendered easy alignment and superposition of the backbones of both proteins, with a RMSD value of 0.9 Å, and resulted in a modeled backbone with relatively few steric clashes (Fig. 2A). When we superposed the TaOMT2 structure onto 1KYZ, but omitted the region close to the SAH binding site, the RMSD fell to 1.1 Å. This indicates that the SAH binding area contributes significantly to the overall similarity of the two backbones, but that the remainder of the model is still in very good agreement with the complete structure. The high similarity of the relative locations of their putative active sites (Fig. 2B), as well as their secondary structures (Additional file 3), attest to the high precision of the proposed model. In addition, all of the amino acid residues neighboring the substrate- and SAM/SAH binding sites are conserved, except for one residue located near the substrate binding site, where Val309 in TaOMT2 is replaced by Ile316 in MsCOMT (Additional file 3), a conservative substitution as well.

### SAM/SAH binding

**Figure 3:**
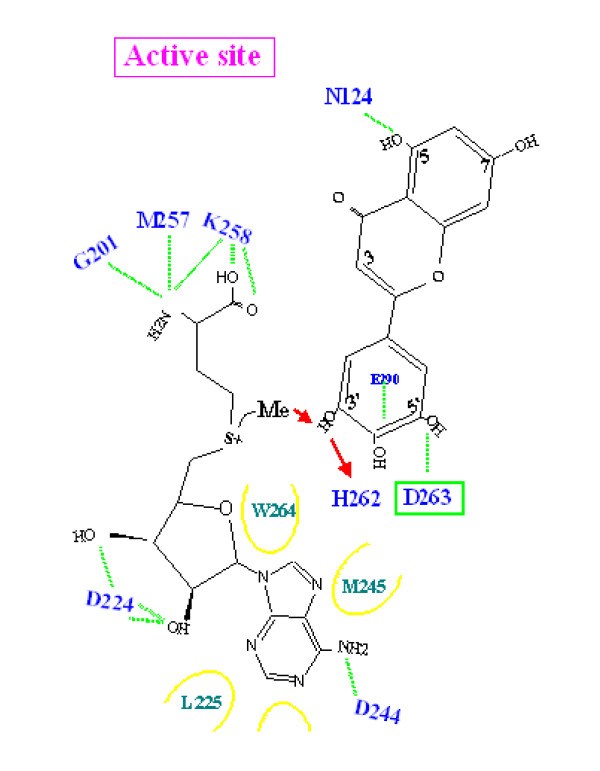
**Schematic view of the active site of TaOMT2 showing the residues involved in binding and catalysis**.

TaOMT2 mediates the transfer of SAM methyl group to tricetin B-ring hydroxyl groups in a sequential manner, resulting in three different methylated derivatives (Fig. 1) and SAH, as products. All residues neighboring the SAM/SAH binding pocket in TaOMT2 are quite conserved. These include Asp199, Asp224, Asp244, Gly201, Lys258, Leu225, Met245 and Trp264. Other residues, including Phe246, Gly203, Ile260, Ser177, Thr207, Trp259, Val200, Met257 and Val228, lie within 4 Å from SAH-S. Like all other SAM-dependent OMTs, SAM/SAH binding is mediated by means of an extensive network of hydrogen bonds and van der Waals interactions that sequester SAM and position its methyl group near tricetin hydroxyl groups. In our TaOMT2 structural model, SAM is bound in a manner where the methyl center is flanked by two structural arms, methionine and ribose-adenine (Fig. 3). The carboxylate group of SAM forms H-bonds with Lys258, and the terminal amino group participate in an H-bond with Gly201, Met257 and Lys258. The ribose hydroxyls are constrained by H-bonds to Asp224, whereas the exocyclic amino group of the adenine ring forms a H-bond with Asp244. The heterocyclic adenine ring is sequestered by a number of hydrophobic residues, with Met245 and Leu225 bracketing the ring, and Trp264 and Phe246 involved in a favorable edge-to-face interaction (Fig. 3).

### Substrate binding by TaOMT2

Based on molecular modeling experiments and manual docking of each of tricetin, selgin and tricin into the model, several H-bonds can be observed within 1.53 to 2.61Å from the ligands and the functional groups of neighboring residues, and within 4.92 to 6.56Å from the catalytic site SAH-S (Table 1). It is apparent that Asp263 plays an important role in binding of the three substrates, since its γ-carboxyl group forms H-bonds with the 3'-hydroxyl group of tricetin, 4'- and 5'-hydroxyls of selgin and 4'-hydroxyl group of tricetin (Fig. 3). The γ-carboxyl groups of Glu290 and Glu322 constrain the B-ring of the substrate through H-bonds with the respective 4'- and 5'-hydroxyl groups of tricetin. The δNH_2 _of Asn124 and the backbone of Gly305 align the flavonoid A- and C-rings in a favorable position for substrate binding through an H-bonding network (Table 1).

**Table 1:** Modeling data for TaOMT2*

Ligand	Methylated product	H-bond (Å)	Distance from SAH-S(Å)
Tricetin	3'OH→3'OMe	-Asp263γCOO^- ^- 3'OH → 1.53	4.92
		-His262δNH - 5'OH →2.08	
		-Glu322δCOO^- ^- 5'OH → 2.11	
		-Asn124δNH_2 _-5O → 1.74	
Selgin	5'OH→5'OMe	-Gly305CO - 7OH → 2.10	5.72
		-Asn124δNH_2 _- 4O →1.87	
		-Asn124δO - 5OH → 2.0	
		-Trp259CO - 4'OH → 2.61	
		-Asp263γCOO^- ^- 5'OH → 2.30	
		-Asp263γCOO^- ^- 4'OH → 2.05	
Tricin	4'OH→4'OMe	-Gly305CO-7OH → 1.92	6.56
		-Asn124δNH_2 _-4O → 1.60	
		-Asn124δO-5OH → 2.13	
		-Asp263γCOO^-^-4'OH → 1.76	

*Tricetin, selgin, and tricin were docked manually into TaOMT2 using FlexX Single Receptor Module in Sybyl, and the H-bond distances were measured between the ligand hydroxyls and functional groups of neighboring residues as well as the catalytic site SAH-S.

### Characterization of mutant proteins

In order to confirm the importance of the putative residues involved in substrate binding (Table 1), they were subjected to site-directed mutagenesis and the expressed mutant proteins were affinity-purified before assaying for enzyme activity (Table 2). Regardless of the differences observed in their expression levels (Additional file 4A), the mutant proteins were quite stable up to 4-6 weeks when stored at 4°C in the assay buffer containing 10% glycerol.

**Table 2:** Significance of the putative residues of TaOMT2 involved in binding ans/or catalysis and changes in the properties of their mutant proteins^1^

Wild type	Significance	Mutant	**Enzyme activity**^**2**^				Properties of mutant proteins
				
residues		proteins	(%)	**K**_**m**_	**V**_**max**_	**K**_**cat**_**/K**_**m**_	
**Control**			100	59.5	110	74	
**D263**	Important residue for substrate binding; forms H-bonds with All OH groups **of **tricetin	**D263E**	4.01				Severe loss of activity is due to a conflict between the catalytic His262-imidazole group and Glu-CH_2_
		**D263I**	0.08				Ile263 can not form a H-bond with 3'-OH group
		**D263N**	84.3^3^	128.2	20.3	63	Slight decrease in activity due to a decreased electronegativity of Asn-N compared to Asp-O, that affects charge transfer to tricetin-OH groups
**E290**	H-bonds with tricetin 4'-OH; forms an H-bonding network with neighboring residues, esp. E290-COO^- ^and H262-backbone-NH	**E290I**	1.7				Loss of activity is due to the fact that Ile can not form a H-bond with the 4'-OH of tricetin
		**E290Q**	0.06				This mutation results in a more extensive H-bonding that hinders charge transfer and affects B-ring flexibility
**W259**	H-bonds with selgin 4'-OH and forms a H-bonding network with neighboring residues	**W259A**	79.2^3^	131.0	17.02	5.20	Ala can maintain the H-bonding network between Trp259, Glu290 and His262, wheras Tyr cannot
		**W259Y**	49.1^3^	170.1	9.90	2.33	
**E322**	H-bonds with tricetin 5'-OH.	**E322I**	54.3^3^	193.17	30.56	6.33	Loss of charge or a change in the side chain affects H-bonding with the neighboring residues, especially His262
		**E322Q**	40.3				
**G305**	H-bonds with selgin 7-OH; important residue for substrate positioning	**G305S**	63.3^3^	118.41	21.65	7.31	Change in polarity is less effective than chain length on catalytic activity.
		**G305A**	0.14				Loss of activity due to loss of H-bonding with the amide group of the neighboring Asn348
**N124**	H-bonds with O-4/O-5 of all substrates in order to orient them to the most favorable position	**N124I**	1.8				Resuled in a decreased substrate binding but not protein folding. Both mutations disrupt H-bonding with 5-OH group of tricetin
		**N124Q**	4.1				
**H262**	Putative catalytic base involved in deprotonation of tricetin hydroxyl groups	**H262R**	0.01				Resulted in almost complete loss of protein expression; all mutant proteins lack imidazole ring that is critical for proton flow among His262, Asp263 and the substrate
		**H262L**	0.96				
		**H262F**	1.06				

^1^Site directed mutagenesis and preparation of mutant proteins were conducted as described in the Methods section.

^2^Enzyme activity with tricetin as substrate is expressed in terms of % relative activity as compared with wild-type protein, K_m _values (μM), V_max _(pkat.mg^-1^; pkat, the catalytic activity that raises the reaction rate by one pmol.s^-1^), and catalytic efficiency (K_cat_/K_m _nM^-1^.s^-1^), respectively.

^3^HPLC analysis of the enzyme reaction products showed tricin as the predominant product, with a trace amount of trimethyltricetin (see Additional Fig. 4B).

Replacement of Asp263 with either glutamic acid or isoleucine resulted in mutant proteins that exhibited severe loss of activity, indicating its critical role in substrate binding (Table 2). Likewise, substitution of His262 with arginine, leucine or phenylalanine abolished the catalytic activity of their mutant proteins. In fact, mutation of the His residue resulted in almost complete loss of protein expression and enzyme activity (Additional file 4A and Table 2). These results indicate the necessity of the imidazole ring for electron flow between Asp263 and His262. Kinetic analyses of mutant proteins with significant relative enzyme activity exhibited 2- to 3-fold lower affinity for tricetin, 70 to 90% reduced reaction velocity and 80 to 95% lower catalytic efficiency compared to the wild-type protein (Table 2). Taken together, these results indicate that changes in the H-bond network, charge transfer and/or size of the target residue have considerable effects on substrate binding and, consequently, the catalytic activity of the mutant proteins. However, it is interesting to note that HPLC analysis of the enzyme reaction products of those mutant proteins showed no significant differences in the product ratios between the wild-type and mutants, where tricin always constituted the predominant enzyme reaction product, with a trace of trimethyltricetin, but no selgin as would be expected (Additional Fig. 4B). Furthermore, mutant proteins with significant OMT activity can methylate selgin to tricin, and the latter to trace amounts of trimethyltricetin (data not shown), thus maintaining the sequential methylation characteristic of the wild-type protein.

### Proposed reaction mechanism for TaOMT2

**Figure 4:**
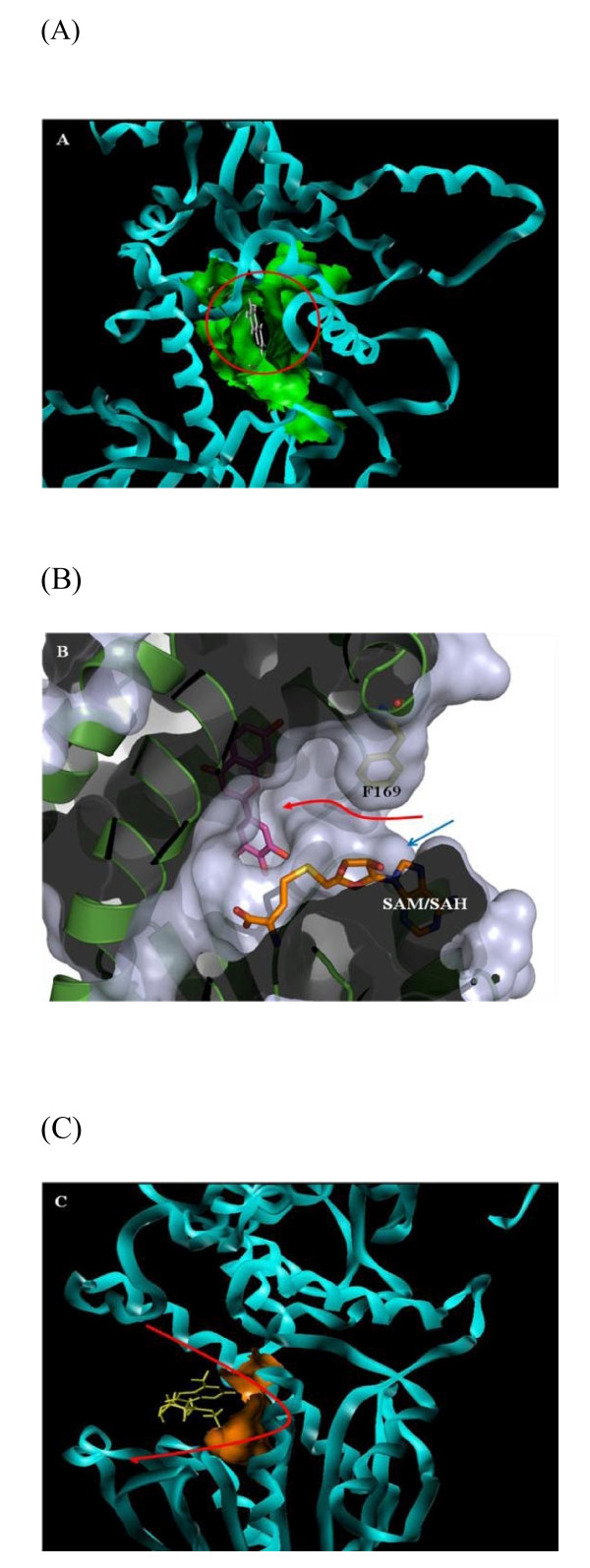
**Architecture of the substrate binding pocket of TaOMT2**. A, The substrate binding pocket with bound tricetin is shown inside the red circle. The residues neighboring the binding site are shown as green spheres; B, The entrance for the substrate, tricetin (red arrow) and the co-substrate, SAM (blue arrow) into the binding pocket. The substrate passes through a tunnel to reach the partly-buried pocket; C, The red parabola indicates SAM/SAH binding site. SAH is shown in yellow color and the residues neighboring the binding site are shown as orange spheres.

**Figure 5:**
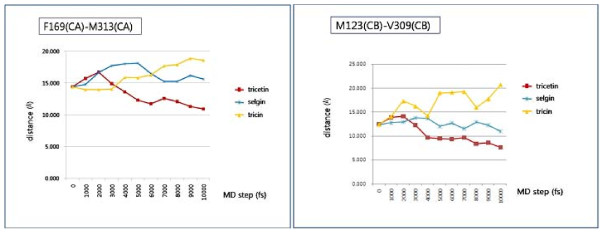
**Molecular dynamics calculations of the size of the active site occupied by the substrates: tricetin, selgin and tricin, as measured between two pairs of the indicated amino acid residues across the site**.

We propose His262 as the catalytic base for deprotonation of the substrate hydroxyl groups, through proximity and orientation effects [26] with the participation of Asp263 (Fig. 3). In fact, the γ-carboxyl group of Asp263 forms H-bonds with each of the hydroxyl groups to be methylated (Table 1). In addition to the negatively charged binding surface contributed by Glu290, Glu322, Gly305 and Asp263, the latter residue serves as a suitable active site residue because of its low pKa value, the dominant effect and stability of its negative charge and its favorable charge interactions with different ligands [27]. Furthermore, the structural model reveals that a portion of the flavonoid active site is buried inside the protein (Fig. 4A), and can only be reached by the substrate through a tunnel (Fig. 4B). It can accommodate the substrates tricetin, selgin, and tricin, and allows for the rotation/re-orientation of the methylated intermediates within the same active site, as determined by MD calculations (Fig. 5) and space filling models that indicated no conflict with neighboring amino acids after 90° rotation of the docked selgin or tricin (data not shown). In contrast with the tight active site, the large open-entrance of SAM/SAH binding site (Fig. 4C) allows for free entry of SAM and exit of SAH during successive methylations.

**Figure 6:**
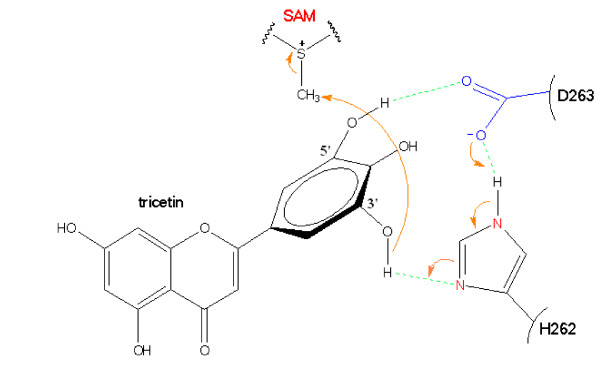
**Hypothetical mechanism for methylation of tricetin by TaOMT2**. 3'-Methylation involves an electron transfer from the γ-carboxyl group of Asp263 to the imidazole ring of His262. The electron-enriched group becomes the nucleophile that attacks the methyl group of SAM to give rise to the first methylated product, selgin.

**Figure 7:**
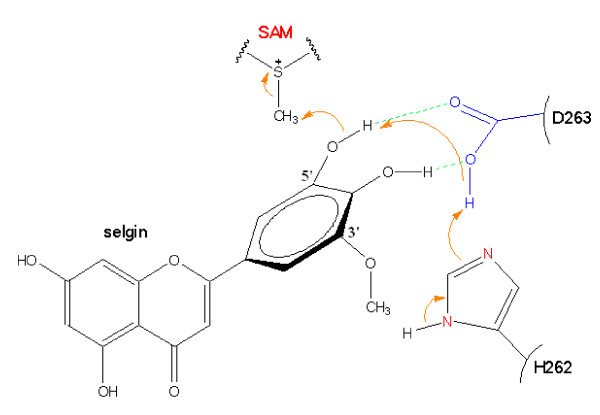
**Hypothetical mechanism for methylation of selgin by TaOMT2.** In contrast with 3'-methylation, electron transfer for methylation of the 5'-hydroxyl group proceeds from water molecules within the reaction centre to the imidazole ring of His262, and further to Asp263 giving rise to tricin.

Stepwise methylation of tricetin involves deprotonation of its 3'-hydroxyl group by the neighboring His262-Asp263 pair, followed by a nucleophilic attack of the SAM-methyl group resulting in the formation of selgin (Fig. 6). Re-orientation of selgin in the substrate binding pocket, and possible conformational change of the residues involved in substrate binding (Table 1), allows further methylation of the 5'-position to give rise to tricin (Fig. 7). As for the 4'-hydroxyl group, it appears to be the least favored position for methylation [4], although it seems feasible both structurally and mechanistically. In fact, when tricin is docked in the binding site, it forms H-bonds with N124, D263 and G305 (Additional file 5); the same amino acid residues involved in binding of tricetin and selgin (Table 1), indicating that tricin does not leave the binding pocket during sequential methylation. However, the fact that further methylation of tricin constitutes *ca*. < 5% of total methylation (Additional file 4B), may be due to the weak binding affinity of the enzyme to this substrate, steric hindrance of the bulky methyl group, or the competitive inhibition of the enzyme reaction by the final product, trimethyltricetin (unpublished data).

### Substrate specificity of TaOMT2

In contrast with the SAM/SAH binding site which is conserved among all SAM-dependent OMTs [14], the residues neighboring the other substrate binding site may be as variable as the compounds preferred by their OMTs [3]. Phenylpropanoids, the preferred substrates for MsCOMT, are structurally similar to tricetin B-ring and its 3-C tail, the preferred substrate of TaOMT2, as would be 5HFA to selgin (Fig. 1). It was surmised, therefore, that the only change among the residues neighboring the substrate binding sites of both proteins, Val309 in TaOMT2 to Ile316 in MsCOMT (Additional file 3), may be responsible for the substrate preference of both proteins. In fact, replacement of Val309 by isoleucine in TaOMT2 resulted in a mutant protein,V309I that exhibited a 15-fold higher affinity and a 4-fold increased catalytic efficiency for 5HFA over tricetin, as substrates (Table 3). On the other hand, the wild type *Medicago truncatula *MtCOMT, an ortholog of MsCOMT with 98% sequence identity as would be expected, exhibits a higher binding affinity and extremely elevated reaction velocity and catalytic efficiency for 5HFA than tricetin. However, the change of Ile316 to valine resulted in a mutant protein, I316V with 67-fold higher catalytic efficiency for tricetin, albeit with a 1.5-fold lower value for 5HFA, as compared to the wild type protein (Additional file 6). This indicates that mutation of a single amino acid, Val309 in TaOMT2 altered its substrate preference from tricetin to 5HFA. The comparable change of Ile316 to valine in MtCOMT significantly increased its turnover for tricetin, but it remained a functional COMT. This is further corroborated by the molecular modeling data which show that, in the wild type TaOMT2 the isopropyl group of Val309 is far (1.8 Å) from, and does not conflict with, either the A- or C-rings of tricetin (Additional file 7A), but the van der Waals clouds (not shown) of the isobutyl group of isoleucine in V309I are closer (0.8 Å) to the A- and C-rings of tricetin (Additional file 7B), that do not favor binding of the latter substrate. However, because of its small size, 5HFA resides loosely in the binding site of the wild type enzyme (Additional file 7C), which explains its low affinity and reduced turnover of 5HFA compared with tricetin. In the mutant V309I, the isobutyl group of Ile309 is large enough to stabilize 5HFA in the binding pocket (Additional file 7D), and results in a higher substrate affinity and a significant catalytic efficiency as compared to tricetin (Table 3). Taken together, these results clearly demonstrate that Val309 in TaOMT2 is responsible for substrate selectivity, and that a single amino acid change is sufficient to alter its substrate preference.

**Table 3:** Kinetic parameters of wild type TaOMT2 and mutant V309I for tricetin and 5HFA as substrates^a^

Substrate	**K**_**m **_**(μM)**	**V**_**max **_**(pkat/mg)**	**K**_**cat/Km **_**(nM**^**-1**^**. s**^**-1**^)
**Wild type**			
Tricetin	59.51 ± 0.79	110 ± 7.0	74
5HFA	270.98 ± 11.51	210 ± 6.0	31
**V309I**			
Tricetin	101.08 ± 9.68	35 ± 8.0	13
5HFA	18.24 ± 1.72	59 ± 2.0	130

^a^The affinity purified recombinant proteins (1.8 μg) were incubated with 5.0 to 80 μM of the indicated substrates, 1 mM of AdoMet containing 125 nCi of the [^3^H] label for 15 min at 30°C, and the activity in the products was determined as described in the Methods section. The data are averages of three determinations ± SE; pkat, the catalytic activity that raises the reaction rate by one pmol.s^-1^.

## Discussion

A 3-D structural model of wheat flavone OMT, TaOMT2, was generated based on the crystal structure of caffeic acid/5-hydroxyferulic acid MsCOMT [16]. The high amino acid sequence identity, superposition of the backbones and the conservation of residues near the active sites (Additional file 3) provided the basis for a model to study the structure of this protein.

In contrast with other plant OMTs which mediate single methyl transfers, TaOMT2 catalyzes the sequential methylation of tricetin, by virtue of the unique architecture and disposition of its active sites (Fig. 3 and Figs. 4A to 4C). The fact that selgin does not accumulate neither *in planta *(unpublished data) nor *in vitro *enzyme assays [[4] and this work] indicates that selgin, the first methylated intermediate of tricetin methylation, does not leave the active site until completion of sequential methylation. Such a mechanism excludes channeling of the methylated intermediates from one active site to another, and is in agreement with a random enzyme reaction mechanism [28]. The stepwise methylation of tricetin starts at the 3'-hydroxyl group which is the preferred (*meta*) position for methylation because of its highest negative electron density [29], as previously shown with the classical examples: rat liver catechol OMT [19] and lignin monomers COMT [16], followed by methylation at the 5'-position, through re-orientation of the first methylated intermediate, selgin. However, the significantly low level of methylation at the 4'-hydroxyl group of tricin (Additional file 4B) may be explained by (a) the weak binding affinity of the enzyme for tricin (data not shown), (b) the low negative electron density of this *para *hydroxyl group [29], (c) the steric hindrance caused by introduction of a bulky methyl group into the 4'-position of tricin, and/or (d) the competitive inhibition of the enzyme reaction by its final product, 3',4',5'-trimethyltricetin (ca < 5 μM) (unpublished data). In fact, the results of enzyme assays and HPLC analysis of the reaction products analysis support these assertions [4,28].

The fact that a single amino acid residue, Val309 in TaOMT2 and Ile316 in MsCOMT, determines the preference for their respective substrates, tricetin and 5HFA (Table 3) is remarkable. Such single amino acid polymorphism [30-32] may define the evolutionary differences between the two closely related phenylpropanoid and flavonoid OMTs, which resulted in mis-annotation of several members of the latter OMT family [13]. Several monocotyledonous flavone OMTs, as well as the Arabidopsis flavonol OMT1 (AtOMT1), share this common residue (valine), whereas it is replaced by either leucine or isoleucine in three COMTs: TaOMT4, MsCOMT and MtOMT1 (Additional file 1). Val is a small aliphatic residue and has one less methylene group than either leucine or isoleucine. It is located near the gate of the substrate binding pocket. Replacement of valine with isoleucine or leucine alters the volume of the binding pocket, thus changes the substrate preference of the enzyme. Furthermore, the natural occurrence of tricin in the forage crops, *M. sativa *[33] and *M. truncatula *[34], represents an example of the competition between both groups of OMTs involved in the methylation of lignin monomers [35] and the flavone, tricetin.

Tricin has been credited for its multifunctional properties and health promoting effects [36], including potent inhibition of expression and activity of cyclooxygenase enzymes, growth inhibition of human malignant breast tumor cells and colon cancer cells [37,38] and reducing the numbers of intestinal adenomas [39], among others. The structure-function relationships of TaOMT2 reported here provide the basis for the enzymatic synthesis of tricin. Furthermore, the molecular model indicates that both Met313 and Asn317 lie within 3 to 4Å from the 4'-hydroxyl group of tricetin (data not shown), that may be involved in the 4'-*O*-methylation step. It will be interesting to investigate whether mutations of these two residues can alter the ratio of tricetin methylation products towards the metabolic engineering and optimization of tricin production in wheat [36].

## Conclusions

TaOMT2 catalyzes the sequential methylation of the flavone tricetin. Substrate binding is mediated by an extensive H-bond network and van der Waals interactions which sequester both the substrate and co-substrate. Methylation is proposed to proceed by deprotonation of the hydroxyl groups via the His262-Asp263 pair, followed by the nucleophilic attack of the SAM-methyl group within the same active site. The sequence of methylation starts at the 3'-hydroxyl group, followed by the 5'-, then the 4'-hydroxyls through re-orientation of intermediates and possible conformational changes of the surrounding residues. Val309 defines the preference of TaOMT2 for its substrate, tricetin.

## Abbreviations

5HFA: 5-hydroxyferulic acid; OMT: *O*-methyltransferase; SAH: *S*-adenosyl-L-homocysteine; SAM: *S*-adenosyl-L-methionine

## Authors' contributions

RKI and JAK designed the project; EL, YP and YL constructed the 3-D model; JMZ and FKS performed the mutagenesis experiments and analyzed the mutant proteins; RKI drafted the manuscript and all co-authors participated in its editing.

## Supplementary Material

Additional file 1 **Partial amino acid sequence alignment of eight graminaceous and three dicotyledonous OMTs**. The amino acid sequences were obtained from GenBank with Accession numbers listed in the order shown in the figure: TaOMT2 *Triticum aestivum *(ABB03907); TaOMT1 *Triticum aestivum *(AAP23942); TaOMT4 *Triticum aestivum *(EF423611) HvOMT1 *Hordeum vulgare *(BI956358); OsOMT1 *Oryza sativa *(DQ530257); SbCOMT *Sorghum bicolor *(AY217766); SoCOMT *Saccharum officinarum *(AJ231133); ZmOMT1 *Zea mays *(DR811764); AtOMT1 *Arabidopsis *(U70424); MsCOMT *Medicago sativa *(AAB46623); MtCOMT *Medicago truncatula *(AW686202). Note that the putative residues neighboring the substrate binding site (purple color) and the residue putatively involved in catalysis (red color) are strictly conserved. The putative residue defining substrate specificity of plant OMTs is in yellow color. Click here for file

Additional file 2 **Primers used to generate *TaOMT2 *mutants**. Mutated codons are underlined with lowercase letters indicating a base change from the wild-type sequence. Click here for file

Additional file 3 **Amino acid sequence alignment of *Triticum aestivum *flavone *O*-methyltransferase (TaOMT2) and *Medicago sativa *caffeic acid/5-hydroxyferulic acid *O*-methyltransferase (MsCOMT) - α-Helices (magenta) and ß-sheets (blue) depict the residues that form the secondary structures of both proteins. **Green stars indicate the putative residues involved in substrate binding, and the magenta star indicates that involved in catalysis. The putative residues involved in substrate preference of both OMTs are boxed. Click here for file

Additional file 4 **Characterization of mutant proteins**. A, SDS-PAGE of recombinant wild type TaOMT2 (1) and some mutant proteins: W259A (2), D263I (3), H262L (4), V309I (5) and N124I (6), represented by equal amounts of the solubilized pellets; B, HPLC profiles of the enzyme reaction products of the wild type and W259A proteins assayed with tricetin as the substrate: 1, Tricin; 2, Trimethyltricetin. Other mutants exhibited HPLC profiles similar to that of W259A. Click here for file

Additional file 5 **Residues neighboring tricin binding site of TaOMT2. **N124, D263, and G305 form H-bonds with tricin. These residues are also involved in binding of the two other substrates. Click here for file

Additional file 6 **Kinetic parameters of the wild type MtCOMT and mutant I316V for tricetin and 5HFA as substrates**. Click here for file

Additional file 7 **The role of Val309 in substrate preference of TaOMT2**. A, in the wild type TaOMT2, the isopropyl group of Val309 is far (1.8 Å) from and does not conflict with either the A- or C-rings of tricetin (brown color); B, in the mutant V309I, the van der Waals clouds of the isobutyl group of Ile309 (not shown) are close (0.8 Å) to and conflict with the A- and C-rings of tricetin; C, because of its small size 5HFA can reside in the binding site with two orientations, whereas in the wile type, the isopropyl group of Val309 is small enough to allow the flexibility of 5HFA inside its binding site; D, in the mutant V309I, the isobutyl group of Ile309 is large enough to stabilize 5HFA inside its binding site. Click here for file

## References

[B1] Ibrahim RK Anzellotti D The enzymatic basis of flavonoid biodiversity Rec Adv Phytochem 2003 37 1 36 full_text

[B2] Lam KC Ibrahim RK Behdad B Dayanandan D Structure, function, and evolution of plant *O*-methyltransferases Genome 2007 50 1001 1013 10.1139/G07-077 18059546

[B3] Ferrer JL Austin MB Stewart C Jr Noel JP Structure and function of enzymes involved in the biosynthesis of phenylpropanoids Plant Physiol Biochem 2008 46 356 470 10.1016/j.plaphy.2007.12.009 18272377 PMC2860624

[B4] Zhou JM Gold ND Martin VJ Wollenweber E Ibrahim RK Sequential *O*-methylation of tricetin by a single gene product in wheat Biochim Biophys Acta 2006 1760 1115 1124 16730127 10.1016/j.bbagen.2006.02.008

[B5] Ridgway RD Vance DE Kinetic mechanism of phosphoethanolamine *N*-methyltransferase J Biol Chem 1988 263 16864 16871 3182819

[B6] Vance JE Vance DE Phospholipid biosynthesis in mammalian cells Biochem Cell Biol 2004 82 113 128 10.1139/o03-073 15052332

[B7] Charron JB Breton G Danyluk J Muzac I Ibrahim RK Sarhan F Molecular and biochemical characterization of a cold-regulated phosphoethanolamine *N*-methyltransferase from wheat Plant Physiol 2002 129 363 373 10.1104/pp.001776 12011366 PMC155899

[B8] Trievel RC Flynn EM Houtz RK Hurley JH Mechanism of multiple lysine methylation by the SET domain Rubisco LSMT Nature Struct Biol 2003 10 545 552 10.1038/nsb946 12819771

[B9] Qian W Wang X Manzur K Farooq SA Zeng L Wang R Zhou MM Structural insights of the specificity and catalysis of a viral histone H3 Lysine27 methyltransferase J Mol Biol 2006 359 86 96 10.1016/j.jmb.2006.03.006 16603186

[B10] De Luca V Ibrahim RK Enzymatic synthesis of polymethylated flavonols in *C. americanum*, I. Partial purification and some properties of *S*-adenosyl-L-methionine: flavonol 3-, 6-, 7- and 4' -*O*-methyltransferases Arch Biochem Biophys 1985 238 596 605 10.1016/0003-9861(85)90205-X 3994393

[B11] De Luca V Ibrahim RK Enzymatic synthesis of polymethylated flavonols in *C. americanum*, II. Substrate interaction and product inhibition studies of flavonol 3-, 6- and 4' -*O*-methyltransferases Arch Biochem Biophys 1985 238 606 618 10.1016/0003-9861(85)90206-1 3994394

[B12] Zhou JM Fukushi Y Wang XF Ibrahim RK Characterization of a novel *O*-methyltransferase gene in rice Nat Prod Commun 2006 1 981 984 10.1080/14786410600921532

[B13] Zhou JM Fukushi Y Wollenweber E Ibrahim RK Characterization of two *O*-methyltransferase-like genes in barley and maize Pharmceut Biol 2008 46 26 34 10.1080/13880200701729745

[B14] Martin JL McMillan FM The *S*-adenosylmethionine-dependent methyltransferase fold Curr Opin Struct Biol 2002 12 783 793 10.1016/S0959-440X(02)00391-3 12504684

[B15] Zubieta C He XZ Dixon RA Noel JP Structures of two natural product methyltransferases reveal the basis for substrate specificity in plant *O*-methyltransferases Nature Struct Mol Biol 2001 8 271 279 10.1038/85029 11224575

[B16] Zubieta C Kota P Ferrer JL Dixon RA Noel JP Structural basis for the modulation of lignin monomer methylation by caffeic acid/5-hydroxyferulic acid 3/5- *O*-methyltransferase Plant Cell 2002 14 1265 1277 10.1105/tpc.001412 12084826 PMC150779

[B17] Zubieta C Ross JR Koscheski P Yang Y Pichersky E Noel JP Structural basis for substrate recognition in the salicylic acid carboxyl methyltransferase family Plant Cell 2003 15 1704 1716 10.1105/tpc.014548 12897246 PMC167163

[B18] Rossmann MG Moras D Olsen KW Chemical and biological solution of a nucleotide-binding protein Nature 1974 250 194 199 10.1038/250194a0 4368490

[B19] Vidgren J Svensson LA Lilijas A Crystal structure of catechol *O*-methyltransferase Nature 1994 368 354 358 10.1038/368354a0 8127373

[B20] Ferrer JL Zubieta C Dixon RA Noel JP Crystal structure of alfalfa caffeoyl coenzyme A 3-*O*-methyltransferase Plant Physiol 2005 137 1009 1017 10.1104/pp.104.048751 15734921 PMC1065401

[B21] Coward JK Salvatore F Chemical mechanisms of methyl transfer reactions: comparison of methylases with nonenzymic 'model reactions' The Biochemistry of Adenosylmethionine 1977 Columbia University Press, NewYork 127 144

[B22] Schubert HL Blumenthal RM Cheng X Many paths to methyltransfer: a chronicle of convergence Trends Biochem Sci 2003 28 329 335 10.1016/S0968-0004(03)00090-2 12826405 PMC2758044

[B23] Chan WL Lin YC Zhang WH Tang PL Szeto YS One step synthesis of polyhydroxyflavones from hydroxyacetophenones and benzaldehydes Heterocycles 1996 43 551 556 10.3987/COM-95-7287

[B24] Yang H Ahn JH Ibrahim RK Lee S Lim Y The three-dimensional structure of *Atabidopsis thaliana O*-methyltransferase predicted by homology-based modelling J Mol Graphics Modell 2004 23 77 87 10.1016/j.jmgm.2004.02.001 15331056

[B25] Lineweaver H Burk D The determination of enzyme dissociation constants J Am Chem Soc 1934 56 658 666 10.1021/ja01318a036

[B26] Takusagawa F Fujioka M Spies A Schowen RL Sinnott IM *S*-Adenosylmethionine (AdoMet)-dependent methyltransferases Comprehensive Biological Catalysis: A mechanistic reference 1998 Academic Press, London 1 30

[B27] Richardson JS Richardson DC Fasman GD Principles and patterns of protein conformation Prediction of Protein Structure and the Principles of Protein Conformation 1989 Plenum Press, New York 1 98

[B28] Kornblatt JA Zhou JM Ibrahim RK Structure-activity relationships of wheat flavone *O*-methyltransferase - a homodimer of convenience FEBS J 2008 275 2255 266 10.1111/j.1742-4658.2008.06377.x 18397325

[B29] Pople JA Beveridge DL Approximate molecular orbital theory 1970 Mc-Graw Hill, New York

[B30] Gang DR Lavid N Zubieta C Chen F Beuerie T Lewinsohn E Noel JP Pichersky E Characterization of phenylpropene *O*-methyltransferases from sweet basil: Facile change of substrate specificity and convergent evolution within a plant OMT family Plant Cell 2002 14 505 519 10.1105/tpc.010327 11884690 PMC152928

[B31] Scalliet G Piola F Douady CJ Réty S Raymond O Baudino S Bordji K Bendahmane M Dumas C Cock JM Hugueney P Scent evolution in Chinese roses Proc Natl Acad Sci USA 2008 105 5927 5932 10.1073/pnas.0711551105 18413608 PMC2311369

[B32] Nogouchi A Horikawa M Fukui Y Fukuchi-Mizutan M Luchi-Okada A Ishiguro M Kiso Y Nakayama T Ono E Local differentiation of sugar donor specificity of flavonoid glycosyltransferase in Lamiales Plant Cell 2009 21 1556 1572 10.1105/tpc.108.063826 19454730 PMC2700533

[B33] Stochmal A Simonet AM Macias FA Oleszek W Alfalfa (*Medicago sativa *L.) flavonoids. 2. Tricin and chrysoeriol glycosides from aerial parts J Agric Food Chem 2001 49 5310 5314 10.1021/jf010600x 11714321

[B34] Kowalska I Stochmal A Kapusta I Janda B Pizza C Piacente S Olfszek W Flavonoids from barrel medic (*Medicago truncatula*) aerial parts J Agric Food Chem 2007 55 2645 2665 10.1021/jf063635b 17348681

[B35] Chen F Reddy MS Temple S Jackson I Shadle G Dixon RA Multi-site genetic modulation of monolignol biosynthesis suggests new routes for formation of syringyl lignin and wall-bound ferulic acid in alfalfa (*Medicago sativa *L.) Plant J 2006 48 113 124 10.1111/j.1365-313X.2006.02857.x 16972868

[B36] Zhou JM Ibrahim RK Tricin--a potential multifunctional nutraceutical Phytochem Rev 2010

[B37] Hudson EA Dinh PA Kokubun T Simmonds MS Gescher AJ Characterization of potentially chemopreventive phenols in extracts of brown rice that inhibit the growth of human breast and colon cancer cells Cancer Epidemiol Biomarkers Prevention 2000 9 1163 1170 11097223

[B38] Cai H Hudson EA Mann P Verschoyle RD Greaves P Manson MM Steward WP Gescher AJ Growth-inhibitory and cell cycle-arresting properties of the rice bran constituent, tricin in human-derived breast cancer cells *in vitro *and in nude mice *in vivo* Br J Cancer 2004 91 1364 1371 10.1038/sj.bjc.6602124 15316567 PMC2410014

[B39] Cai H Al-Fayez M Tunstall-Platton S Greaves P Steward WP Gescher AJ The rice bran constituent tricin potently inhibits cyclooxygenase enzymes and interferes with intestinal carcinogenesis in *Apc*^*Min *^mice Mol Cancer Ther 2005 4 1287 1292 10.1158/1535-7163.MCT-05-0165 16170019

