# Supplementary material for: Structure-function relationships of wheat flavone O-methyltransferase: Homology modeling and site-directed mutagenesis
Source: BMC Plant Biol. 2010 Jul 29;10:156. doi: 10.1186/1471-2229-10-156 (PMC3017781; doi:10.1186/1471-2229-10-156)
Supplement: Additional file 1 — Partial amino acid sequence alignment of eight graminaceous and three dicotyledonous OMTs. The amino acid sequences were obtained from GenBank with Accession numbers listed in the order shown in the figure: TaOMT2 Triticum aestivum (ABB03907); TaOMT1 Triticum aestivum (AAP23942); TaOMT4 Triticum aestivum (EF423611) HvOMT1 Hordeum vulgare (BI956358); OsOMT1 Oryza sativa (DQ530257); SbCOMT Sorghum bicolor (AY217766); SoCOMT Saccharum officinarum (AJ231133); ZmOMT1 Zea mays (DR811764); AtOMT1 Arabidopsis (U70424); MsCOMT Medicago sativa (AAB46623); MtCOMT Medicago truncatula (AW686202). Note that the putative residues neighboring the substrate binding site (purple color) and the residue putatively involved in catalysis (red color) are strictly conserved. The putative residue defining substrate specificity of plant OMTs is in yellow color. [file 1471-2229-10-156-S1.DOC]

**Additional file 1 - Partial amino acid sequence alignment of eight graminaceous and three dicotyledonous OMTs.**

The amino acid sequences were obtained from GenBank with Accession numbers listed in the order shown in the figure: TaOMT2 *Triticum aestivum* (ABB03907); TaOMT1 *Triticum aestivum* (AAP23942); TaOMT4 *Triticum aestivum* (EF423611) HvOMT1 *Hordeum vulgare* (BI956358); OsOMT1 *Oryza sativa* (DQ530257); SbCOMT *Sorghum bicolor* (AY217766); SoCOMT *Saccharum officinarum* (AJ231133); ZmOMT1 *Zea mays* (DR811764); AtOMT1 *Arabidopsis* (U70424); MsCOMT *Medicago sativa* (AAB46623); MtCOMT *Medicago truncatula* (AW686202). Note that the putative residues neighboring the substrate binding site (purple color) and the residue putatively involved in catalysis (red color) are strictly conserved. The putative residue defining substrate specificity of plant OMTs is in yellow color.

**TaOMT2** MGS-------IAAGADEDACMYALQLVSSSILPMTLKNAIELGLLETLMAAG-------GKFLTPAEVAAKLPSA-ANP-EAPDMVDRMKFLTPAEVAAKLPSA-ANP-KF

**TaOMT1** MGSTAADMAA---SADEEACMYALQLVSSSILPMTLKNAIELGLLETLVAAG-------GKLLTPAEVAAKLPST-ANP-AAADMVDRMKLLTPAEVAAKLPST-ANP-KL

**TaOMT4** MGS----------TADEEACMFALQLGSSSILPMTLKNAIELGLLDTLVAAD-------GKLLSPAELAAKLPST-ANP-AAPDMVDRMKLLSPAELAAKLPST-ANP-KL

**HvCOMT** MGS----IAA---GADEDACMYALQLVSSSILPMTLKNAIELGLLETLMSAG-------GKFLTPAEVAAKLPST-ANP-EAPDMVDRMKFLTPAEVAAKLPST-ANP-KF

**OsOMT1** MGSTAADMAA---AADEEACMYALQLASSSILPMTLKNAIELGLLETLQSAAVAGGGGKAALLTPAEVADKLPSK-ANP-AAADMVDRMALLTPAEVADKLPSK-ANP-AL

**SbCOMT** MGSTAEDVAA---VADEEACMYAMQLASSSILPMTLKNALELGLLEVLQKDA-------GKALAAEEVVARLPVAPTNP-AAADMVDRMKALAAEEVVARLPVAPTNP-KA

**SoCOMT** MGSTAEDVAA---VADEEACMYAMQLASASILPMTLKNALELGLLEVLQAEAPA-----GKALAPEEVVARLPVAPTNP-DAADMVDRMKALAPEEVVARLPVAPTNP-KA

**ZmOMT1** MGSTAGDVAA---VVDEEACMYAMQLASSSILPMTLKNAIELGLLEVLQKEAGGG----KAALAPEEVVARMPAAPGDPAAAAAMVDRMAALAPEEVVARMPAAPGDPAAA

**AtOMT1** MGSTAETQLTPVQVTDDEAALFAMQLASASVLPMALKSALELDLLEIMAKN--------GSPMSPTEIASKLPTK--NP-EAPVMLDRISPMSPTEIASKLPTK--NP-SP

**MsCOMT** MGSTGETQITPTHISDEEANLFAMQLASASVLPMILKSALELDLLEIIAKAGP------GAQISPIEIASQLPTT--NP-DAPVMLDRMAQISPIEIASQLPTT--NP-AQ

**MtOMT1** MGSTGETQITPTHISDEEANLFAMQLASASVLPMVLKSALELDLLEIIAKAGP------GAQISPIEIASQLPTT--NP-EAPVMLDRIAQISPIEIASQLPTT--NP-AQ

**262 290 309**

**TaOMT2** DLPHVISEAPPFPGVTHVGGDMFQKVP-SGDAILMKWILHDWSDEHCATLLKNCYDALPAHG-KVVLVECILPVNPEATPKAQGVFHVDMIMLAHNPHG-KVVLVECILPH

**TaOMT1** DLPHVISEAQPFPGVTHVGGDMFQKVP-SGDAILMKWILHDWSDEHCATLLKNCYDALPAHG-KVVLVECILPVNPEATPKAQGVFHVDMIMLAHNPHG-KVVLVECILPH

**TaOMT4** DLSHVISEAPPFPGVTHVGGDMFQKIP-SGDTILMKWILHDWSDEHCATLLKNCYDALPTHG-KVMLVECILPVNPEATPKAQGGFHLDMIMLAHNPHG-KVMLVECILPH

**HvCOMT** DLPHVISEAPAFPGVTHIGGDMFQKVP-SGDAILMKWILHDWSDEHCATLLKNCYDALPAHG-KVVLVECILPVNPEATPEVQGVFHVDMIMLAHNPHG-KVVLVECILPH

**OsOMT1** DLPHVISEAPPFPGVEHVGGDMFASVPRGGDAILMKWILHDWSDEHCARLLKNCYDALPEHG-KVVVVECVLPESSDATAREQGVFHVDMIMLAHNPHG-KVVVVECVLPH

**SbCOMT** DLPHVISEAPPFPGVQHVGGDMFKSVP-AGDAILMKWILHDWSDAHCATLLKNCYDALPEKGGKVIVVECVLPVTTDAVPKAQGVFHVDMIMLAHNPKGGKVIVVECVLPK

**SoCOMT** DLPHVISEAPPFPGVQHVGGDMFKSVP-AGDAILMKWILHDWSDAHCATLLKNCYDALPENG-KVIIVECVLPVNTEAVPKAQGVFHVDMIMLAHNPNG-KVIIVECVLPN

**ZmOMT1** DLPHVISEAPPFLGVRHVGGDMFASVP-AGDAILMKWILHDWSDAHCATLLKNCYDALPENG-KVIVVECVLPVNTEATPKAQGVFHVDMIMLAHNPNG-KVIVVECVLPN

**AtOMT1** NLPHVIEDAPSHPGIEHVGGDMFVSVP-KGDAIFMKWICHDWSDEHCVKFLKNCYESLPEDG-KVILAECILPETPDSSLSTKQVVHVDCIMLAHNPDG-KVILAECILPD

**MsCOMT** DLPHVIEDAPSYPGVEHVGGDMFVSIP-KADAVFMKWICHDWSDEHCLKFLKNCYEALPDNG-KVIVAECILPVAPDSSLATKGVVHIDVIMLAHNPNG-KVIVAECILPN

**MtOMT1** DLPHVIEDAPSYPGVEHVGGDMFVSIP-KADAVFMKWICHDWSDEHCLKFLKNCYEALPDNG-KVIVAECILPVAPDSSLATKGVVHIDAIMLAHNPNG-KVIVAECILPN

**322**

**TaOMT**2 HG-KVVLVECILPVNPEATPKAQGVFHVDMIMLAHNPGGRERYEREFEALAKGAGFAAMKTTYXYANAWAIEFTK-- 356

**TaOMT**1 HG-KVVLVECILPVNPEATPKAQGVFHVDMIMLAHNPGGRERYEREFEALAKGAGFKAIKTTYIYANAFAIEFTK-- 360

**TaOMT**4 HG-KVMLVECILPVNPEATPKAQGGFHLDMIMLAHNPGGKERYEREFEALAKGAGFGAMKTTYIYANTWVIEFTK-- 353

**HvCOM**T HG-KVVLVECILPVNPEATPEVQGVFHVDMIMLAHNPGGRERYEREFEALAKGAGFAAMKTTYIYANAWAIEFTK-- 356

**OsOMT**1 HG-KVVVVECVLPESSDATAREQGVFHVDMIMLAHNPGGKERYEREFRELARAAGFTGFKATYIYANAWAIEFTK-- 368

**SbCOM**T KGGKVIVVECVLPVTTDAVPKAQGVFHVDMIMLAHNPGGRERYEREFRDLAKAAGFSGFKATYIYANAWAIEFIK-- 362

**SoCOM**T NG-KVIIVECVLPVNTEAVPKAQGVFHVDMIMLAHNPGGRERYEREFHDLAKGAGFSGFKATYIYANAWAIEFIK-- 362

**ZmOMT**1 NG-KVIVVECVLPVNTEATPKAQGVFHVDMIMLAHNPGGKERYEREFRELAKGAGFSGFKATYIYANAWAIEFIK-- 364

**AtOMT**1 DG-KVILAECILPETPDSSLSTKQVVHVDCIMLAHNPGGKERTEKEFEALAKASGFKGIKVVCDAFGVNLIELLKKL 363

**MsCOM**T NG-KVIVAECILPVAPDSSLATKGVVHIDVIMLAHNPGGKERTQKEFEDLAKGAGFQGFKVHCNAFNTYIMEFLKKV 365

**MtOMT**1 NG-KVIVAECILPVAPDSSLATKGVVHIDAIMLAHNPGGKERTQKEFEDLAKGAGFQGFKVHCNAFNTYIMEFLKKV 365
