# Supplementary material for: Structure-function relationships of wheat flavone O-methyltransferase: Homology modeling and site-directed mutagenesis
Source: BMC Plant Biol. 2010 Jul 29;10:156. doi: 10.1186/1471-2229-10-156 (PMC3017781; doi:10.1186/1471-2229-10-156)
Supplement: Additional file 2 — Primers used to generate TaOMT2 mutants. Mutated codons are underlined with lowercase letters indicating a base change from the wild-type sequence. [file 1471-2229-10-156-S2.DOC]

**Additional file 2 - Primers used to generate *TaOMT2* mutants**.

Mutated codons are underlined with lowercase letters indicating a base change from the wild-type sequence.

---------------------------------------------------------------------------------------------------------------------

V309IF GCAGGGGGTGTTCCATattGACATGATCATGCTCG

V309IR GAGCATGATCATGTCaatATGGAACACCCCCTGCG

N124IF CTCGCGCTCATGAttCAGGACAAGGTCC

N124IR GACCTTGTCCTGaaTCATGAGCGCGAGC

N124QF TCGCGCTCATGcagCAGGACAAGGTC

N124QR ACCTTGTCCTGctgCATGAGCGCGAG

G305SF GAGGCGACGCCTAAGGCGCAGagcGTGTTCCATGTCGACATGATC

G305SR GATCATGTCGACATGGAACACgctCTGCGCCTTAGGCGTCGCCTC

G305AF CTAAGGCGCAGgcgGTGTTCCATG

G305AR GATTCCGCGTCcgcCACAAGGTAC

E290IF AAGGTGGTGCTCGTGattTGCATCCTGCCTGTG

E290IR ACAGGCAGGATGCAaatCACGAGCACCACCTTG

E290QF CAAGGTGGTGCTCGTGcaaTGCATCCTGCCTGTG

E290QR CACAGGCAGGATGCAttgCACGAGCACCACCTTG

H262LF ATGAAGTGGATCCTCCtgGACTGGAGCGACGAGC

H262LR CTCGTCGCTCCAGTCcaGGAGGATCCACTTCATG

H262RF ATGAAGTGGATCCTCCgtGACTGGAGCGACGAGC

H262RR CTCGTCGCTCCAGTCacGGAGGATCCACTTCATG

H262FF CATGAAGTGGATCCTCtttGACTGGAGCGACGAGC

H262FR GCTCGTCGCTCCAGTCaaaGAGGATCCACTTCATG

E322IF ACAACCCGGGTGGCAGGattTAGGTACGAGAGGGAGTTC

E322IR AACTCCCTCTCGTACCTaatCCTGCCACCCGGGTTGTG

E322QF ACAACCCGGGTGGCAGGcaaAGGTACGAGAGGGAGTTC

E322QR AACTCCCTCTCGTACCTttgCCTGCCACCCGGGTTGTG

W259AF CATCCTCATGAAGgcGATCCTCCACGAC

W259AR TCGTGGAGGATCgcCTTCATGAGGATGG

W259YF CCATCCTCATGAAGTatATCCTCCACGACTGG

W259YR CAGTCGTGGAGGATatACTTCATGAGGATGGC

D263IF AAGTGGATCCTCCACattTGGAGCGACGAGCAC

D263IR TGCTCGTCGCTCCAaatGTGGAGGATCCACTTC

D263EF ATCCTCCAcGAATGGAGCGACGAG

D263ER TCGTCGCTCCAtTCGTGGAGGATC

D263NF AAGTGGATCCTCCACaatTGGAGCGACGAGCAC

D263NR TGCTCGTCGCTCCAattGTGGAGGATCCACTTC

N317DF TCATGCTCGCGCACgACCCGGGTGGCAGG

N317DR CTGCCACCCGGGTcGTGCGCGAGCATGAT

I316VF CAAAAGGTGTGGTTCACgtgGATGCAATCATGTTGGC

I316VR GCCAACATGATTGCATCcacGTGAACCACACCTTTTG

---------------------------------------------------------------------------------------------------------------------
