# Supplementary material for: Structure-function relationships of wheat flavone O-methyltransferase: Homology modeling and site-directed mutagenesis
Source: BMC Plant Biol. 2010 Jul 29;10:156. doi: 10.1186/1471-2229-10-156 (PMC3017781; doi:10.1186/1471-2229-10-156)

**Additional file 3. - Amino acid sequence alignment of *Triticum aestivum* flavone *O*-methyltransferase (TaOMT2) and *Medicago sativa* caffeic acid/5-hydroxyferulic acid *O*-methyltransferase (MsCOMT) -** α-Helices (magenta) and ß-sheets (blue) depict the residues that form the secondary structures of both proteins. Green stars indicate the putative residues involved in substrate binding, and the magenta star indicates that involved in catalysis. The putative residues involved in substrate preference of both OMTs are boxed.


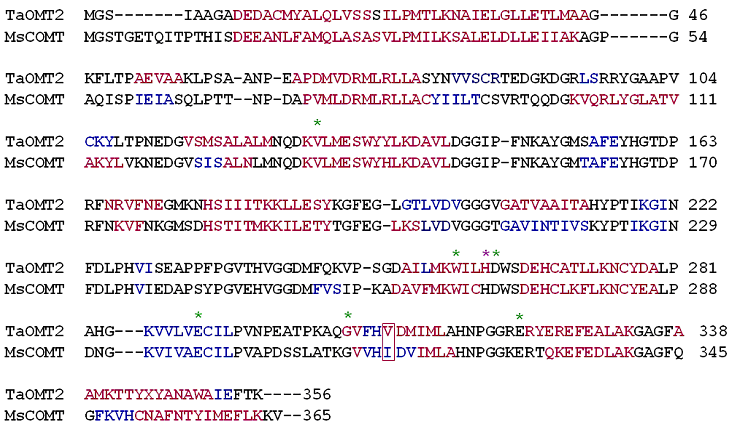

Supplement: Additional file 3 — Amino acid sequence alignment of Triticum aestivum flavone O-methyltransferase (TaOMT2) and Medicago sativa caffeic acid/5-hydroxyferulic acid O-methyltransferase (MsCOMT) - α-Helices (magenta) and ß-sheets (blue) depict the residues that form the secondary structures of both proteins. Green stars indicate the putative residues involved in substrate binding, and the magenta star indicates that involved in catalysis. The putative residues involved in substrate preference of both OMTs are boxed. [file 1471-2229-10-156-S3.DOC]
