# Supplementary material for: Structure-function relationships of wheat flavone O-methyltransferase: Homology modeling and site-directed mutagenesis
Source: BMC Plant Biol. 2010 Jul 29;10:156. doi: 10.1186/1471-2229-10-156 (PMC3017781; doi:10.1186/1471-2229-10-156)
Supplement: Additional file 4 — Characterization of mutant proteins. A, SDS-PAGE of recombinant wild type TaOMT2 (1) and some mutant proteins: W259A (2), D263I (3), H262L (4), V309I (5) and N124I (6), represented by equal amounts of the solubilized pellets; B, HPLC profiles of the enzyme reaction products of the wild type and W259A proteins assayed with tricetin as the substrate: 1, Tricin; 2, Trimethyltricetin. Other mutants exhibited HPLC profiles similar to that of W259A. [file 1471-2229-10-156-S4.doc]

**Additional file 4 - Characterization of mutant proteins (A)**, SDS-PAGE of recombinant wild type TaOMT2 (1) and some mutant proteins: W259A (2), D263I (3), H262L (4), V309I (5) and N124I (6), represented by equal amounts of the solubilized pellets; **(B)**, HPLC profiles of the enzyme reaction products of the wild type and W259A proteins assayed with tricetin as the substrate: 1, Tricin; 2, Trimethyltricetin. Other mutants exhibited HPLC profiles similar to that of W259A.


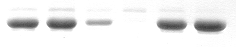


**1 2 3 4 5 6**


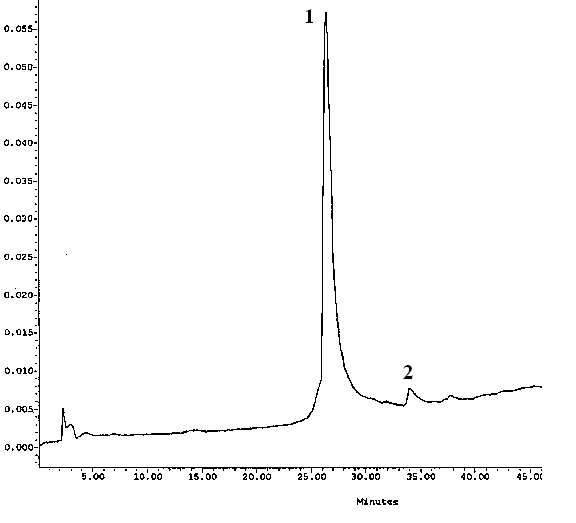

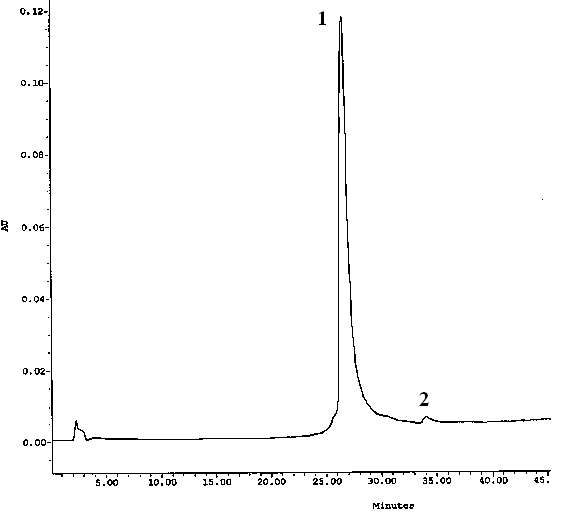


**A**

**B**

**Wild type**

**W259A**
