# Supplementary material for: Structure-function relationships of wheat flavone O-methyltransferase: Homology modeling and site-directed mutagenesis
Source: BMC Plant Biol. 2010 Jul 29;10:156. doi: 10.1186/1471-2229-10-156 (PMC3017781; doi:10.1186/1471-2229-10-156)
Supplement: Additional file 5 — Residues neighboring tricin binding site of TaOMT2. N124, D263, and G305 form H-bonds with tricin. These residues are also involved in binding of the two other substrates. [file 1471-2229-10-156-S5.doc]

**Additional file 5. - Residues neighboring tricin binding site of TaOMT2.** N124, D263, and G305 form H-bonds with tricin. These residues are also involved in binding of the two other substrates.

**
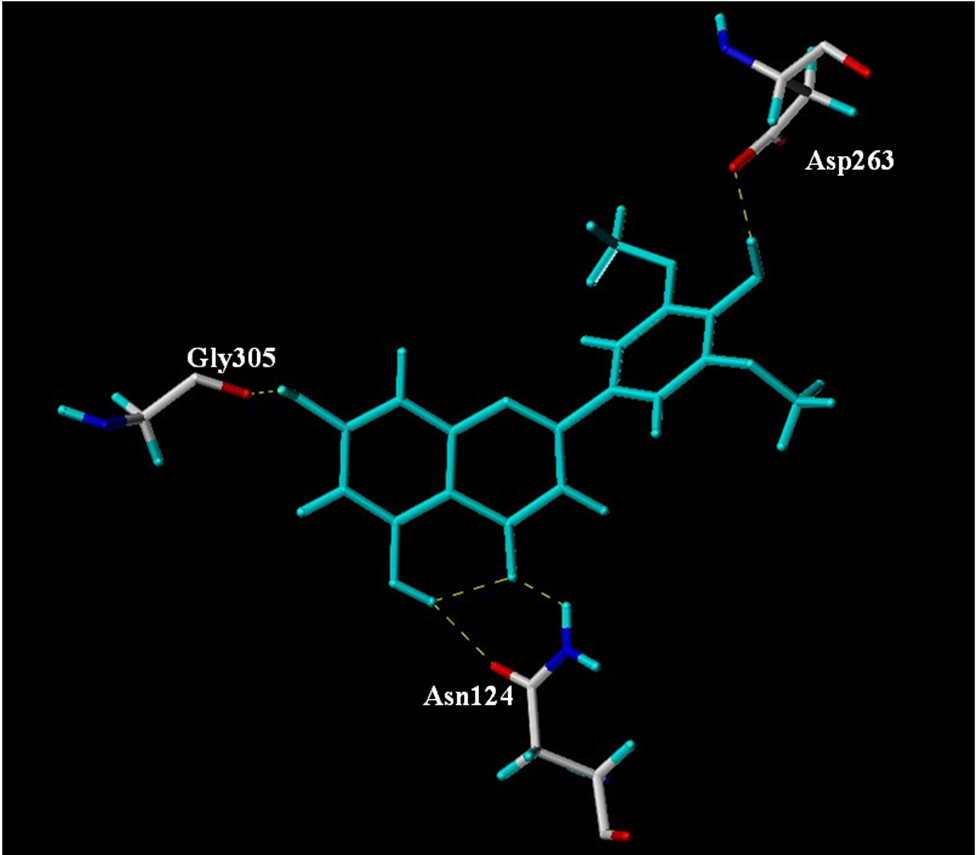
**
