# Supplementary material for: Structure-function relationships of wheat flavone O-methyltransferase: Homology modeling and site-directed mutagenesis
Source: BMC Plant Biol. 2010 Jul 29;10:156. doi: 10.1186/1471-2229-10-156 (PMC3017781; doi:10.1186/1471-2229-10-156)
Supplement: Additional file 6 — Kinetic parameters of the wild type MtCOMT and mutant I316V for tricetin and 5HFA as substrates. [file 1471-2229-10-156-S6.doc]

**Additional file 6 - Kinetic parameters of the wild type MtCOMT and mutant I316V for tricetin and 5HFA as substrates^a^.**

---------------------------------------------------------------------------------------------------------------------

Substrate K_m_  V_max_ K_cat_ / K_m_

(µM) (pkat.mg^-1^) (nM^-1^.s^-1^)

---------------------------------------------------------------------------------------------------------------------

**Wild type**

Tricetin 63.22±9.68 1.5±0.4 0.09

5HFA 26.18±1.97 59±6.0 90

**I316V**

Tricetin 185.90±16.51 28±7.0 6

5HFA 39.16±1.02 59±2.0 60

---------------------------------------------------------------------------------------------------------------------

^a^ The affinity purified recombinant proteins (1.8µg) were incubated with 5.0 to 80µM of the indicated substrates, 1mM of AdoMet containing 125 nCi of the [^3^H] labeled AdoMet, for 15 min at 30ºC, and the activity in the products were determined as described in the Experimental section. The data are averages of three separate determinations± SE.; pkat, the catalytic activity that raises the reaction rate by one pmol.s^-1^.
