# Supplementary material for: Structure-function relationships of wheat flavone O-methyltransferase: Homology modeling and site-directed mutagenesis
Source: BMC Plant Biol. 2010 Jul 29;10:156. doi: 10.1186/1471-2229-10-156 (PMC3017781; doi:10.1186/1471-2229-10-156)
Supplement: Additional file 7 — The role of Val309 in substrate preference of TaOMT2. A, in the wild type TaOMT2, the isopropyl group of Val309 is far (1.8 Å) from and does not conflict with either the A- or C-rings of tricetin (brown color); B, in the mutant V309I, the van der Waals clouds of the isobutyl group of Ile309 (not shown) are close (0.8 Å) to and conflict with the A- and C-rings of tricetin; C, because of its small size 5HFA can reside in the binding site with two orientations, whereas in the wile type, the isopropyl group of Val309 is small enough to allow the flexibility of 5HFA inside its binding site; D, in the mutant V309I, the isobutyl group of Ile309 is large enough to stabilize 5HFA inside its binding site. [file 1471-2229-10-156-S7.doc]

**Additional file 7.** - **The role of Val309 in substrate preference of TaOMT2**. **A,** in the wild type TaOMT2, the isopropyl group of Val309 is far (1.8 Å) from and does not conflict with either the A- or C-rings of tricetin (brown color); **B,** in the mutant V309I, the van der Waals clouds of the isobutyl group of Ile309 (not shown) are close (0.8 Å) to and conflict with the A- and C-rings of tricetin; **C,** because of its small size 5HFA can reside in the binding site with two orientations, whereas in the wile type, the isopropyl group of Val309 is small enough to allow the flexibility of 5HFA inside its binding site; **D,** in the mutant V309I, the isobutyl group of Ile309 is large enough to stabilize 5HFA inside its binding site.

**
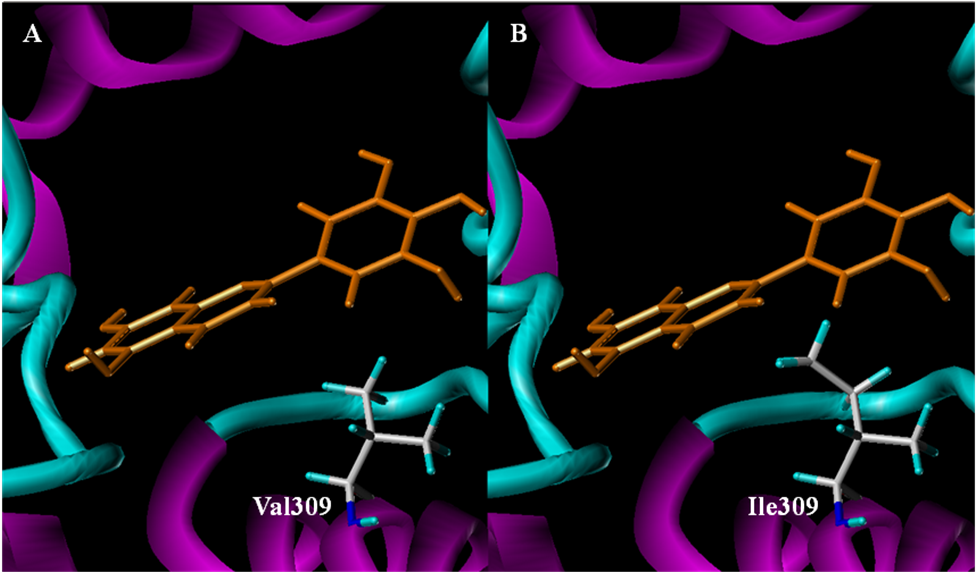
**

**
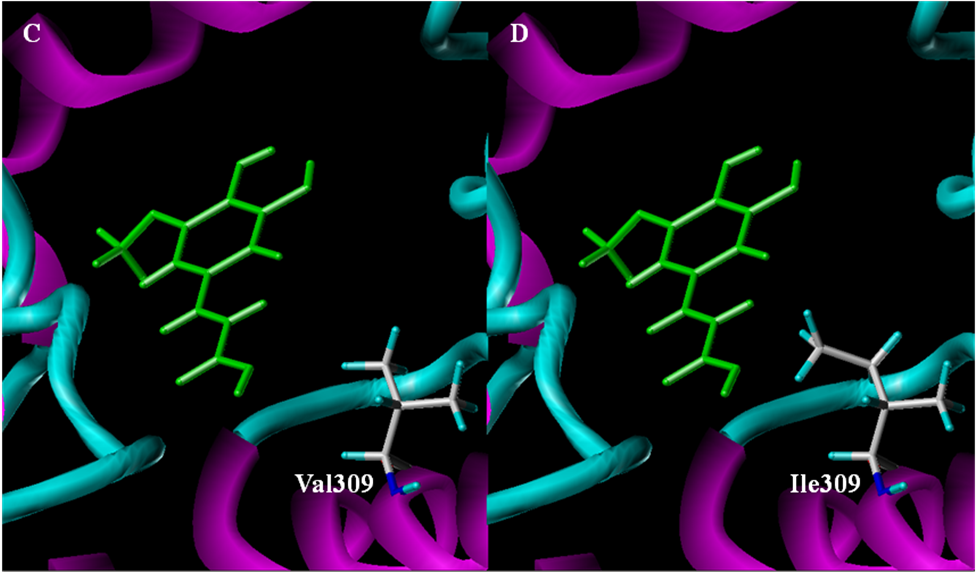
**
